# Supplementary material for: Impact of the Gram-Negative-Selective Inhibitor MAC13243 on In Vitro Simulated Gut Microbiota
Source: Pharmaceuticals (Basel). 2022 Jun 9;15(6):731. doi: 10.3390/ph15060731 (PMC9229071; doi:10.3390/ph15060731)
Supplement: Supplementary file 1 [file pharmaceuticals-15-00731-s001.zip › pharmaceuticals-1715549-supplementary.pdf]

Supplementary Material

Impact of the Gram-Negative-Selective Inhibitor MAC13243 on In Vitro Simulated Gut Microbiota

Frida Svanberg Frisinger [1,†], Mattia Pirolo [1,†], Duncan YK Ng [1,2], Xiaotian Mao [3], Dennis Sandris Nielsen [3] and Luca Guardabassi [1,*]

[1] Department of Veterinary and Animal Sciences, Faculty of Health and Medical Sciences, University of Copenhagen, 1870 Frederiksberg, Denmark; frida.frisinger@sund.ku.dk (F.S.F.); mapi@sund.ku.dk (M.P.); yong.kai.ng@sund.ku.dk (D.Y.K.N.)
[2] Department of Bacteria, Parasites and Fungi, Statens Serum Institut, 2300 Copenhagen, Denmark
[3] Department of Food Science, Faculty of Science, University of Copenhagen, 1870 Frederiksberg, Denmark;
xiaotian.mao@food.ku.dk (X.M.); dn@food.ku.dk (D.S.N.)
* Correspondence: lg@sund.ku.dk
† These authors contributed equally to this work.

Faecal sample collection and processing

Faecal samples were collected from two healthy human volunteers not exposed to antibiotics in the 6 months prior to sampling. Ethical permission for the collection of these samples was waived by the Ethical Committee of the Capital Region of Denmark (registration number H-20028549). The samples were shipped on ice to the laboratory immediately after collection, kept at -20 °C, and processed within 24 hours upon shipment, as described by Anjum et al. [20]. Briefly, the faeces were weighed, and an equal amount (wt/vol) of 20% glycerol/0.1 M PBS solution was added prior to homogenization in a stomacher for 2 × 60 seconds. The resulting faecal suspensions were aliquoted in 2-ml cryotubes and frozen at -80 °C. Immediately before the start of the CoMiniGut experiment, each suspension was thawed and diluted at 1:5 with 0.1 M PBS at pH 5.6.

MIC testing

The faecal suspension from each donor was serially diluted 1:10 in saline and spotted on MacConkey agar (Sigma-Aldrich). Five colonies were randomly collected from each donor and subjected to bacterial identification by MALDI-TOF (Vitek MS, bioMerieux). The minimal inhibitory concentration (MIC) of MAC13243 (range 0.5-256 mg/L) was evaluated on all isolates by broth microdilution in Mueller Hinton Broth (Oxoid) following CLSI recommendations [17] . *Escherichia coli* ATCC 25922 was included as a reference control [9].

CoMiniGut experiment

CoMiniGut is an in vitro system that simulates the colon passage of the human gut [10]. The CoMiniGut prototype consists of a climate box with five parallel single-vessel (5 ml volume) stirred anaerobic reactor units, which are pH-monitored and -controlled [10]. Each vessel was inoculated under anaerobic conditions with 10% (vol/vol) of the faecal sample diluted in basal colon medium (BCM) [10]. For each donor, four fermentations were run in parallel and included three concentrations of MAC13243 (16, 32, and 64 mg/L) and an untreated negative control. Over the experimental time course (8 hours), the pH increased from 5.7 to 6.0 to simulate the proximal colon. One millilitre of the faecal suspension was collected immediately prior to drug inoculation, and after 4 and 8 h of incubation at 37 °C. Of this 1 ml, 900 µl were immediately frozen at -80 °C, and the remaining 100 µl were used for counting the total aerobically culturable bacteria and coliforms. An additional sample was collected for the enumeration of viable cells after 24 hours (experimental endpoint).

Total aerobically culturable bacteria and coliforms count

The total number of aerobically culturable bacteria and coliforms were counted on Brain Heart Infusion (BHI) agar (Oxoid) and MacConkey agar (Sigma-Aldrich), respectively. Briefly, 100 µl of faecal suspension that was collected prior to the addition of antimicrobials, and after 4, 8, and 24 hours of fermentation, was serially diluted 1:10 in saline, and 5 µl were spotted in triplicates on agar plates. The plates were incubated at 37 °C for 24 hours prior to colony counting.

Microbial DNA extraction and 16S rRNA gene sequencing

The total microbial DNA was extracted with the QIAamp Fast DNA Stool Mini Kit (QIAGEN) with a bead-beating step to facilitate cell lysis using the Pathogen Lysis Tubes S (QIAGEN), as previously described [18]. DNA was also extracted from sterile BMC, which served as the extraction control.

The V3-V4 region of the 16S rRNA gene was amplified from the extracted DNA using Quick-16S Primer Set V3-V4 within the Quick-16S NGS Library Prep Kit (Zymo Research).. Amplification was carried out using the LightCycler 96 System (Roche Life Science). Illumina adapters were added to the partial 16S rRNA gene-specific amplicons, which were further processed using the Quick-16S NGS Library Prep Kit (Zymo Research). Amplicons were pooled in equimolar ratios and subsequently purified with the Select-a-Size MagBead (Zymo Research). The DNA concentration of the sequence library was determined using the Qubit quantification system (Life Technologies). Each step of the library preparation was performed using ZymoBIOMICS DNase/RNase Free Water (Zymo Research). A negative control was sequenced to verify that contamination did not occur during the amplification and sequencing. Furthermore, the sequencing performance was validated using a synthetic mock community of eight known organisms employing the ZymoBIOMICS Microbial Community DNA Standard (Zymo Research). Sequencing was performed on Illumina MiSeq (2 × 300 bp paired-end reads) using the MiSeq Reagent Kit v3 (600 cycles; Illumina) according to the manufacturer's instructions.

Quantification of total bacteria by qPCR

The total bacteria were quantified by quantitative PCR (qPCR) using 16S rRNA primers 338F (ACTCCTACGGGAGGCAG) and 530R (GTATTACCGCGGCTGCTG), as previously described [19]. Real-time PCR assays were performed on the LightCycler 96 System (Roche Life Science) in 20 µl reactions using the FastStart Essential DNA Green Master Mix (Roche Life Science) with the

additions of each primer to a final concentration of 0.5 µM. The cycling conditions were as follows: 2 min at 95 °C; 40 cycles of 20 s at 95 °C and 60 s at 61 °C; and a melt curve step from 60 °C to 95 °C.

A qPCR standard curve was created with 10-fold dilutions of the full-length 16S rRNA gene amplified from *Escherichia coli* ATCC 25922 using primers 8F (AGAGTTTGATCCTGGCTCAG) and 1492R (GGTTACCTTGTTACGACTT) and the DreamTaq Green PCR Master Mix (ThermoFisher Scientific). The PCR was carried out in the ProFlex PCR System (Applied Biosystems) under the following conditions: 2 min at 95 °C; 30 cycles of 30 s at 95 °C, 30 s at 52 °C, and 90 s at 72 °C; 5 min at 72 °C. The PCR products were purified using the GeneJET PCR Purification Kit (ThermoFisher Scientific) according to the manufacturer's instructions. The PCR products were visualised on a 1% agarose gel containing ethidium bromide and GeneRuler 1 kb Plus DNA Ladder (ThermoFisher Scientific) to ensure fragment lengths approximating 1484 bp. The DNA concentration was determined by the Qubit quantification system (Life Technologies). The 16S rRNA gene copy number was calculated using the following equation: copy number = $(C/X) \times 0.912\times10^{12}$, where C is the DNA concentration measured (ng/µL) and X is the PCR fragment length (bp).

The number of target copies in each sample was calculated using the following equation: copy number = [10^(-1/S)]^(I-Ct), where S is the slope of the log-linear part of the standard curve, I is the intercept of the standard curve, and Ct is the cycle threshold of the sample.

Sequencing data processing and analyses

The sequencing data were processed using DADA2 v1.14.1 [21]. as implemented in R v3.6.1. The optimal filtering and trimming parameters were identified using FIGARO v3.0 [28]. Forward reads were truncated at 285 bp and reverse reads at 245 bp, with a maximum expected error of 3 for both paired reads and with no ambiguous bases allowed. Forward and reverse 5′ 16S primers were trimmed from the reads using the trimLeft function in DADA2 to trim 16 bp from the forward reads and 24 bp from the reverse reads. Exact amplicon sequence variants (ASVs) were inferred for each sample independently using the DADA2 sample inference algorithm and the estimated error models (learnErrors function). Full, denoised sequences were obtained by merging the inferred forward and reverse reads, and subsequently removing chimeric sequences. A taxonomy table was assembled by assigning taxonomy to each ASV using the Silva taxonomic database v138.1 [27] for DADA2 at the lowest taxonomic rank up to the genus level. Species-level assignment was performed with DADA2::addSpecies, using exact matching to assign species where possible. Likely contaminants

were identified and removed using decontam v.1.12.0 [22] based on a combined contaminant identification method (frequency and prevalence methods). A phyloseq object was constructed from the ASV and taxonomy tables in R using phyloseq v1.30.0 [25] for subsequent analyses. The full version of the sequence table was used for alpha diversity analysis assessed based on the Chao1 index, as implemented in phyloseq. A non-metric multidimensional scaling (nMDS) plot based on the Bray–Curtis dissimilarity matrix was used to visualise differences in the overall microbial community compositions between donors. The statistical significance of these differences was calculated by permutational multivariate analysis of variance (PERMANOVA) using the Adonis function from the vegan v.2.5.7 package. Plots were created with ggplot2 v3.3.0.

LolA phylogenetic analysis

The LolA protein sequences of the main Gram-negative bacteria (reference genome) were retrieved from the NCBI database. The alignment of the protein sequence was performed using MEGA X v10.0.5 [23] and a phylogenetic tree was constructed using IQ-TREE version v.1.5.5 [26] with the best model found by the implemented ModelFinder and bootstrap analysis using 1000 replicates. The tree was visualized using iTOL v6.1.2 [24].
